# Multifaceted In Silico and DFT-Based Analysis of 2‑Aminosuccinic Acid on Ferroptosis-Related Targets

**DOI:** 10.1021/acsomega.6c02875

**Published:** 2026-09-09

**Authors:** Mehmet Hanifi Kebiroğlu, Serap Yalcin Azarkan, Nevin Çankaya

**Affiliations:** † Malatya Turgut Ozal University, Darende Bekir Ilicak Vocational School, Department of Opticianry, Malatya 44700, Turkey; ‡ Department of Medical Pharmacology, Kırsehir Ahi Evran University, Faculty of Medicine, Kırşehir 40100, Turkey; § Usak University, Vocational School of Health Services, Usak 64200,Turkey

## Abstract

Metabolic reprogramming and redox imbalances in cancer cells play a significant role in tumor growth and development of treatment resistance. In recent years, ferroptosis, a type of programmed cell death characterized by iron-dependent lipid peroxidation, has emerged as a promising target for cancer therapy. Amino acid transport, glutathione (GSH) homeostasis, and antioxidant defense mechanisms are critically important in the regulation of ferroptosis. In this study, the interactions of 2-aminosuccinic acid, involved in amino acid metabolism, with key molecular targets associated with ferroptosis were investigated using an in silico molecular docking approach. Docking analyses revealed that the compound showed significant binding affinities with SLC1A1 (EAAT3), SLC7A11 (xCT), and the GPX4/GSH system. The observed binding energy of −5.0 kcal/mol with SLC7A11 is noteworthy in terms of modulating intracellular cysteine/glutathione balance and regulating ferroptosis sensitivity. These findings suggest that 2-aminosuccinic acid may be a potential molecule capable of influencing ferroptosis in cancer cells via amino acid transport and redox regulation. In addition, the structural, electronic, spectroscopic, and thermodynamic properties of 2-aminosuccinic acid were extensively investigated using density functional theory (DFT) with the 6-311G basis set. The molecule’s geometry was optimized to a stable conformation, and the calculated bond lengths followed values reported in the literature. The HOMO–LUMO energy gap (Δ*E* = 3.308 eV) indicated that the molecule has high kinetic stability and low chemical reactivity. Theoretical FT-IR, ^1^H NMR, and ^13^C NMR spectra confirmed the functional groups, while UV–vis analyses revealed strong absorptions in the 150–200 nm range due to π→π* and n→π* transitions. Molecular electrostatic potential (MEP) maps have shown that oxygen atoms form reactive centers as electron-rich regions. Non-covalent interaction (NCI) and Hirshfeld surface analyses revealed that crystal packing is largely stabilized by O···H and H···O hydrogen bonds. Thermo-chemistry map (TCM) analyses detailed the temperature-dependent behavior of thermodynamic parameters such as heat capacity and entropy in the 300–900 K temperature range. This study presents a holistic approach to both the interactions of 2-aminosuccinic acid with ferroptosis-related molecular targets and its fundamental theoretical properties; the findings suggest that this compound could be a potential candidate for future applications in cancer treatment, ferroptosis-targeted drug design, and medicinal chemistry.

## Graphical abstract

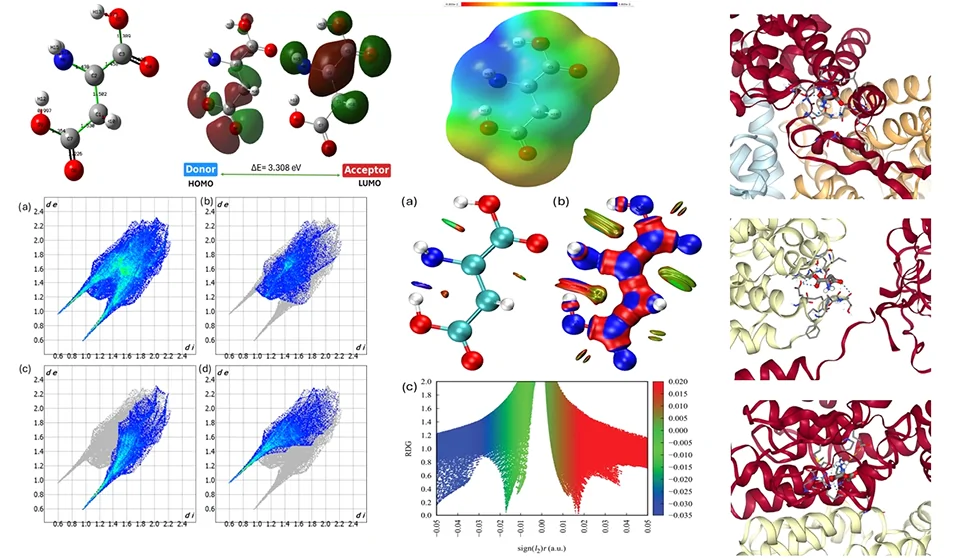

## 1 Introduction

Amino acids, the main building blocks of proteins, play indispensable roles in many biological processes, from enzymatic catalysis and metabolic regulation to cellular signaling and structural support.^1^ Among the 20 canonical amino acids, aspartic acid (also known as 2-aminosuccinic acid or aspartate) occupies a distinct position because of its unique acidic character, conferred by two carboxyl groups in its side chain.^2^ As a nonessential amino acid, it is endogenously synthesized in humans and participates critically in central metabolic pathways, including the urea cycle and gluconeogenesis.^3^ Beyond its metabolic functions, aspartic acid serves as an excitatory neurotransmitter in the central nervous system, contributing to neuroplasticity and cognitive processes.^4^ It also acts as a biosynthetic precursor for other important biomolecules, such as purines, pyrimidines, and amino sugars.^5^ Given this broad biological significance, a deep understanding of the molecular, electronic, and structural properties of aspartic acid is essential for explaining its behavior in physiological contexts and for optimizing its applications in fields ranging from pharmaceutical chemistry to food science and industrial synthesis.^6^ The molecular architecture of 2-aminosuccinic acid featuring an amino group and two carboxyl groups attached to a central carbon backbone endows it with amphoteric properties, allowing it to function as either an acid or a base depending on the pH of its environment.^7^ In both the solid state and aqueous physiological solutions, the molecule predominantly adopts a zwitterionic form, stabilized by an intricate network of intra- and intermolecular hydrogen bonds.^8^ These non-covalent interactions are pivotal in determining its conformational stability, solubility, crystal packing, and recognition by biological receptors.^9^ While experimental techniques such as X-ray crystallography, nuclear magnetic resonance (NMR) spectroscopy, and infrared (IR) spectroscopy have helped to characterize amino acid structures, they often provide limited insight into finer details such as electron density distributions, frontier orbital interactions, and the nuanced role of weak non-covalent forces.^10^ To bridge this gap, computational quantum chemistry approaches, particularly density functional theory (DFT), have emerged as powerful complementary tools.^11^

DFT offers an ideal balance between computational efficiency and accuracy, enabling reliable predictions of molecular geometry, vibrational spectra, NMR chemical shifts, optical properties, and reactivity descriptors.^12^ Key computational analyses such as frontier molecular orbital (FMO) theory provide valuable insights into chemical reactivity and kinetic stability through the energy gap between the highest occupied molecular orbital (HOMO) and the lowest unoccupied molecular orbital (LUMO).^13^ Molecular electrostatic potential (MEP) mapping visualizes charge distribution to identify sites susceptible to electrophilic or nucleophilic attack.^14^ Advanced methods like non-covalent interaction (NCI) analysis, reduced density gradient (RDG) plots, and Hirshfeld surface analysis allow detailed examination of hydrogen bonding and van der Waals interactions that govern molecular stability and crystal packing.^15^

Although several theoretical studies have been conducted on aspartic acid and its derivatives, there remains a need for a unified and comprehensive computational investigation that integrates multiple spectroscopic, electronic, and thermodynamic analyses within a single theoretical framework.^16^ In this context, the present study tries to perform a detailed DFT-based characterization of 2-aminosuccinic acid using the 6-311G basis set.^17^ We systematically explore its optimized geometry, vibrational (FT-IR) and magnetic resonance (NMR) spectra, electronic structure through FMO and density of states (DOS) analyses, and reactivity via MEP mapping.^18^ The work further extends to visualizing non-covalent interactions through NCI and Hirshfeld surface analyses and evaluating thermodynamic behavior across a temperature range.^19^ The findings are expected to provide a strong theoretical reference that supports future experimental work and promotes applications in medicinal chemistry, materials science, and beyond.^20^

Cancer is a complex disease characterized not only by genetic alterations but also by profound changes in cellular metabolism and redox homeostasis.^21^ Cancer cells reprogram antioxidant defenses and amino acid metabolism to support survival and proliferation. Therefore, targeting regulated cell death pathways has emerged as a promising strategy in cancer therapy. Ferroptosis, an iron-dependent form of regulated cell death driven by lipid peroxidation, has attracted considerable attention because of its therapeutic potential in overcoming treatment resistance and improving anticancer efficacy.^22–24^ Recent studies have highlighted ferroptosis-based therapeutic approaches as emerging strategies in precision oncology and nanomedicine applications.^25,26^ Glutathione peroxidase 4 (GPX4) is a key regulator of ferroptosis through detoxification of lipid hydroperoxides, whereas depletion of intracellular glutathione (GSH) promotes ferroptotic cell death.^27^ Since cysteine availability is critical for GSH synthesis, overexpression of the cystine/glutamate antiporter SLC7A11 (xCT) has been associated with ferroptosis resistance and poor prognosis in several cancers.^28^ In addition, SLC1A1 (EAAT3), involved in glutamate and aspartate transport, contributes to amino acid balance and redox regulation, potentially influencing ferroptosis sensitivity.

In this study, molecular docking was used to investigate the interactions of 2-aminosuccinic acid with three ferroptosis-related targets, SLC1A1, SLC7A11, and the GPX4/GSH system, representing amino acid transport, glutathione metabolism, and lipid peroxide detoxification, respectively. The docking findings suggest that 2-aminosuccinic acid may modulate ferroptosis-associated pathways and disrupt the redox homeostasis in cancer cells. These results provide a basis for future *in vitro* and *in vivo* studies of its therapeutic potential.

## 2 Computational Methods

### 2.1 Quantum-Chemical Calculations

All quantum-chemical calculations in this study were performed using the Gaussian 09 software package.^29^ The ground-state molecular geometry of 2-aminosuccinic acid was optimized using DFT with the hybrid B3LYP functional and the 6-311G­(d,p) basis set.^30^ The choice of this level of theory represents a widely accepted and reliable approach for predicting the structural, electronic, and spectroscopic properties of medium-sized organic molecules with a good balance between accuracy and computational cost.^31^ To confirm that the optimized structure corresponds to a true minimum on the potential energy surface (and not a transition state), a subsequent frequency calculation was performed at the same level as theory.^32^ The absence of imaginary vibrational frequencies verified the stability of the optimized conformation.^33^ This frequency analysis also yielded the theoretical infrared (IR) spectrum and fundamental thermochemical data, including zero-point vibrational energy (ZPVE), enthalpy, and Gibbs free energy.^34^ The electronic properties and chemical reactivity of the molecule were investigated through Frontier Molecular Orbital (FMO) analysis.^33^ The energies of the highest occupied molecular orbital (HOMO) and the lowest unoccupied molecular orbital (LUMO) were obtained directly from the single-point energy calculation of the optimized structure.^35^ Key global reactivity descriptors including chemical potential (μ), electronegativity (χ), chemical hardness (η), softness (S), and electrophilicity index (ω) came from these orbital energies using established theoretical formulas.^36^ The density of states (DOS) and total density of states (TDOS) spectra were generated using the Multiwfn program (version 3.8) to visualize the contribution of molecular orbitals to the electronic structure. Theoretical nuclear magnetic resonance (NMR) parameters were calculated using the gauge-independent atomic orbital (GIAO) method at the same B3LYP/6-311G­(d,p) level. The isotropic shielding constants for ^1^H and ^13^C nuclei were converted to chemical shifts (δ in ppm) by referencing to tetramethylsilane (TMS), using the standard equation δ = σ­(TMS) – σ­(sample), where σ­(TMS) is the calculated shielding constant of TMS at the same level of theory. To explore the optical properties, time-dependent DFT (TD-DFT) calculations were conducted at the B3LYP/6-311G­(d,p) level to simulate the ultraviolet–visible (UV–vis) absorption spectrum in the gas phase. The first 20 singlet–singlet excited states were calculated, and the corresponding excitation energies, oscillator strengths (f), and dominant orbital transitions were analyzed. The molecular electrostatic potential (MEP) map was generated from the optimized electron density to visualize regions of positive (nucleophilic attack sites) and negative (electrophilic attack sites) electrostatic potential. Non-covalent interactions (NCIs) were investigated through the non-covalent interaction (NCI) index analysis and visualized using the reduced density gradient (RDG) method, as implemented in the Multiwfn program. The corresponding isosurfaces and 2D RDG scatter plots were generated to distinguish between attractive (e.g., hydrogen bonds), weak interacting (van der Waals), and repulsive interactions. To analyze intermolecular interactions in a crystalline environment, Hirshfeld surface analysis and two-dimensional fingerprint plots were generated using the CrystalExplorer software (version 21.5). This analysis provides a quantitative breakdown of close atomic contacts (O···H, H···H, N···H) contributing to the crystal packing. For the Hirshfeld surface analysis, the experimental CIF file of dl-aspartic acid was obtained from the Crystallography Open Database (COD entry: 2215349). The crystal structure was originally reported by Wang et al. in Acta Crystallographica Section E.^37^ The CIF file was imported into CrystalExplorer 21.5, and the Hirshfeld surface and two-dimensional fingerprint plots were generated to evaluate the intermolecular contacts responsible for the solid-state crystal packing.^38^ Thermodynamic properties including heat capacity at constant volume (*C_v_*), entropy (*S*), and thermal energy (*E*) were calculated as a function of temperature (from 300 to 900 K) using standard statistical thermodynamic formulas based on the vibrational frequencies obtained from the frequency calculation.

### 2.2 Molecular Docking Analyses

To assess the drug’s binding affinities and interaction patterns with important regulatory proteins implicated in cancer-related signaling pathways, molecular docking investigations were conducted. AutoDock 4.2.6 and SeamDock software were used to perform docking simulations with an emphasis on the expected ligand–protein binding sites.^38–43^ For upcoming docking analyses, the target proteins’ crystallographic structures were obtained from the RCSB Protein Data Bank (https://www.rcsb.org/) and stored in PDB format. The three-dimensional structures of SLC1A1 (PDB ID: 6S3Q), SLC7A11 (PDB ID: 7CCS), and GSH (PDB ID: 2LJR) were retrieved from the Protein Data Bank (PDB). Protein preparation was performed using AutoDock Tools (ADT, version 1.5.6). Water molecules and any cocrystallized ligands were removed, polar hydrogen atoms were added, and Kollman charges were assigned to the protein structures. The prepared proteins were subsequently saved in PDBQT format. The structure of 2-aminosuccinic acid was prepared using AutoDock Tools by assigning Gasteiger charges, merging nonpolar hydrogen atoms, and defining rotatable bonds. The ligand was then converted into PDBQT format for docking analysis. Grid maps were generated using AutoGrid. A grid box with dimensions of 100 × 100 × 100 points was defined to encompass the potential binding region of each target protein. Molecular docking simulations were performed using the Lamarckian Genetic Algorithm (LGA) implemented in AutoDock 4.2.6 with default parameters. Multiple docking runs were carried out for each protein–ligand complex, and the resulting conformations were ranked according to their predicted binding free energies (kcal/mol). The docking pose exhibiting the lowest binding energy was selected for further interaction analysis and visualization using Discovery Studio Visualizer. Since molecular docking provides only a theoretical prediction of protein–ligand interactions, the obtained results were interpreted as preliminary computational evidence and should be further validated using additional computational and experimental approaches.

## 3 Results and Discussion

### 3.1 Geometry Optimization

The initial step in the computational characterization of 2-aminosuccinic acid involved the full optimization of its molecular geometry to obtain the most stable ground-state conformation. The optimization was performed using DFT at the B3LYP/6-311G­(d,p) level of theory. The absence of imaginary frequencies in the subsequent vibrational frequency calculation confirmed that the optimized structure corresponds to a true local minimum on the potential energy surface. The optimized molecular structure is presented in Figure 1, illustrating the three-dimensional spatial arrangement of atoms and functional groups. Key geometric parameters, including bond lengths and bond angles, are listed in Table 1. The analysis reveals structural parameters consistent with the expected bonding patterns for an amino acid with both amino and dual carboxylate functionalities. The carbonyl (C=O) bond lengths were calculated as 1.226 and 1.238 Å, which are of typical double-bond character in carboxylic acid groups. The corresponding C–O single bonds in the carboxyl groups were 1.364 and 1.389 Å, respectively. These values align well with standard bond lengths for protonated carboxylic acids and reflect the expected bond order alternation. The C–N bond length, associated with the amino group, was 1.438 Å, consistent with a carbon–nitrogen single bond. The aliphatic C–C bonds in the backbone were calculated to be about 1.502 and 1.530 Å, which fall within the expected range for sp^3^-hybridized carbon atoms. The optimized geometry shows a configuration where the carboxyl and amino groups adopt spatial orientations that help with intramolecular hydrogen bonding, a feature critical for the stability of the zwitterionic form common in solid and aqueous phases. This optimized structure serves as the foundational reference for all subsequent spectroscopic, electronic, and reactivity analyses performed in this study. Although the present DFT calculations provide useful intrinsic information on the structural, electronic, and spectroscopic behavior of 2-aminosuccinic acid, the gas-phase model does not fully reproduce the physiological environment. Aspartic acid is known to predominantly exist in zwitterionic or ionized forms in aqueous and biological media. Solvation can stabilize charge-separated structures and may therefore influence the calculated dipole moment, frontier orbital energies, molecular electrostatic potential distribution, vibrational frequencies, and hydrogen-bonding pattern. The gas-phase results reported here should be regarded as baseline molecular descriptors, but the biological relevance of 2-aminosuccinic acid should be interpreted considering solvent effects and protonation-state equilibria.

**Figure 1:**
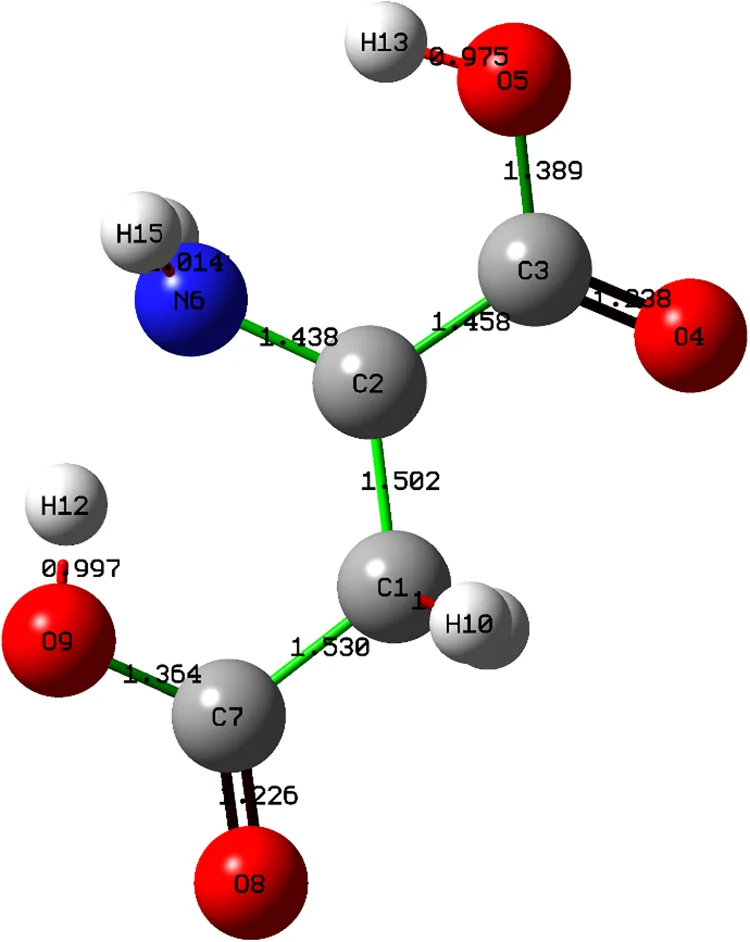
Optimized molecular geometry of 2-aminosuccinic acid obtained at the B3LYP/6-311G­(d,p) level.

**Table 1:** Selected Optimized Geometric Parameters (Bond Lengths in Å) for 2-Aminosuccinic Acid

bond	length (Å)	bond	length (Å)
C1–C2	1.502	C3–O5	1.389
C1–C7	1.530	O5–H13	0.975
C1–H10	1.100	N6–H14	1.014
C1–H11	1.100	N6–H15	1.014
C2–C3	1.458	C7–O8	1.226
C2–N6	1.438	C7–O9	1.364
C3–O4	1.238	O9–H12	0.997

### 3.2 Frontier Molecular Orbital (FMO) Analysis

The frontier molecular orbital (FMO) analysis provides important insights into the chemical reactivity, kinetic stability, and charge-transfer tendencies of a molecule. In this study, the distributions and energies of the highest occupied molecular orbital (HOMO) and the lowest unoccupied molecular orbital (LUMO) for 2-aminosuccinic acid were calculated at the B3LYP/6-311G­(d,p) level. The three-dimensional isosurface plots of HOMO and LUMO are presented in Figure 2. The HOMO, with an energy of −4.352 eV, is predominantly localized over the oxygen atoms of the carboxyl groups and the nitrogen atom of the amino group, suggesting these sites as the primary electron-donating regions of the molecule. But the LUMO, with an energy of −1.044 eV, is mainly distributed across the carbonyl (C=O) π* orbitals and the carbon backbone, designating these as the likely electron-accepting sites. The energy difference between HOMO and LUMO, known as the HOMO–LUMO gap (Δ*E*), is a key indicator of molecular stability and chemical reactivity. The calculated Δ*E* for 2-aminosuccinic acid is 3.308 eV. A relatively large HOMO–LUMO gap such as this suggests that the molecule has high kinetic stability and low chemical reactivity, implying resistance to electronic excitation and charge transfer in ground-state reactions. To further measure the chemical behavior, several global reactivity descriptors came from the frontier orbital energies using established quantum-chemical principles. These parameters are summarized in Table 2.

**Figure 2:**
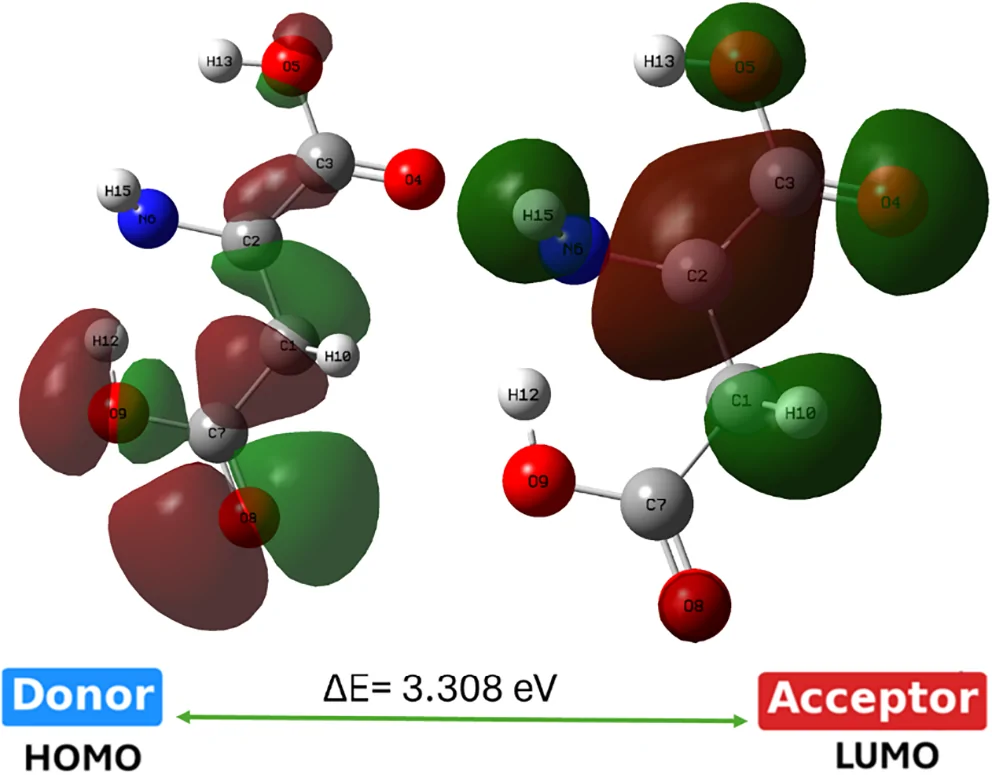
Spatial distributions of the highest occupied molecular orbital (HOMO) and the lowest unoccupied molecular orbital (LUMO) for 2-aminosuccinic acid. The calculated HOMO–LUMO energy gap (Δ*E*) is 3.308 eV.

**Table 2:** Calculated Frontier Orbital Energies and Global Reactivity Descriptors for 2-Aminosuccinic Acid

parameter	symbol	value (eV)	interpretation
HOMO energy	*E*_HOMO_	–4.352	related to electron-donation ability
LUMO energy	*E*_LUMO_	–1.044	related to electron-accepting ability
energy gap	Δ*E*	3.308	indicates high kinetic stability and low chemical reactivity
chemical potential	μ	–2.698	tendency of electrons to escape
electronegativity	χ	2.698	ability to attract electrons
chemical hardness	η	1.654	resistance to transfer charges correlates with stability
chemical softness	*S*	0.302	reciprocal of hardness; indicates polarizability
electrophilicity	ω	2.201	classifies the molecule as a moderate electrophile
electron-accepting	ω^+^	0.738	tendency to accept electrons
electron-donating	ω^–^	14.516	strong tendency to donate electrons

### 3.3 Spectroscopic Analysis

#### 3.3.1 Vibrational Spectroscopic Analysis

Fourier transform infrared (FT-IR) spectroscopy is a fundamental analytical technique for characterizing molecular structures by probing their vibrational modes. The theoretical FT-IR spectrum of 2-aminosuccinic acid was simulated at the B3LYP/6-311G­(d,p) level of theory and is presented in Figure 3. The computed vibrational frequencies and their corresponding assignments for the most typical bands are summarized in Table 3, providing a detailed correlation between the spectral features and the molecular structure. The simulated spectrum shows the typical vibrational pattern of an amino acid with carboxyl and amino functional groups. In the high-frequency region (3500–2500 cm^–1^), a broad and intense absorption band is seen, centered at 3000 cm^–1^. This feature is attributed to the overlapping O–H stretching vibrations of the carboxylic acid groups and the N–H stretching vibrations of the protonated amino group (−NH_3_^+^), which follow the zwitterionic form of the molecule in its ground state. The broadening of this band is typical of strong hydrogen-bonding interactions involving these donors. The most intense and diagnostic peak in the spectrum appears at 1742 cm^–1^, corresponding to the C=O stretching vibration of the carbonyl groups. This strong absorption is typical of the carbonyl functional group and confirms the presence of carboxylic acid moieties. The calculated frequency aligns well with the typical experimental range for protonated carboxylic acids (1710–1750 cm^–1^).

**Figure 3:**
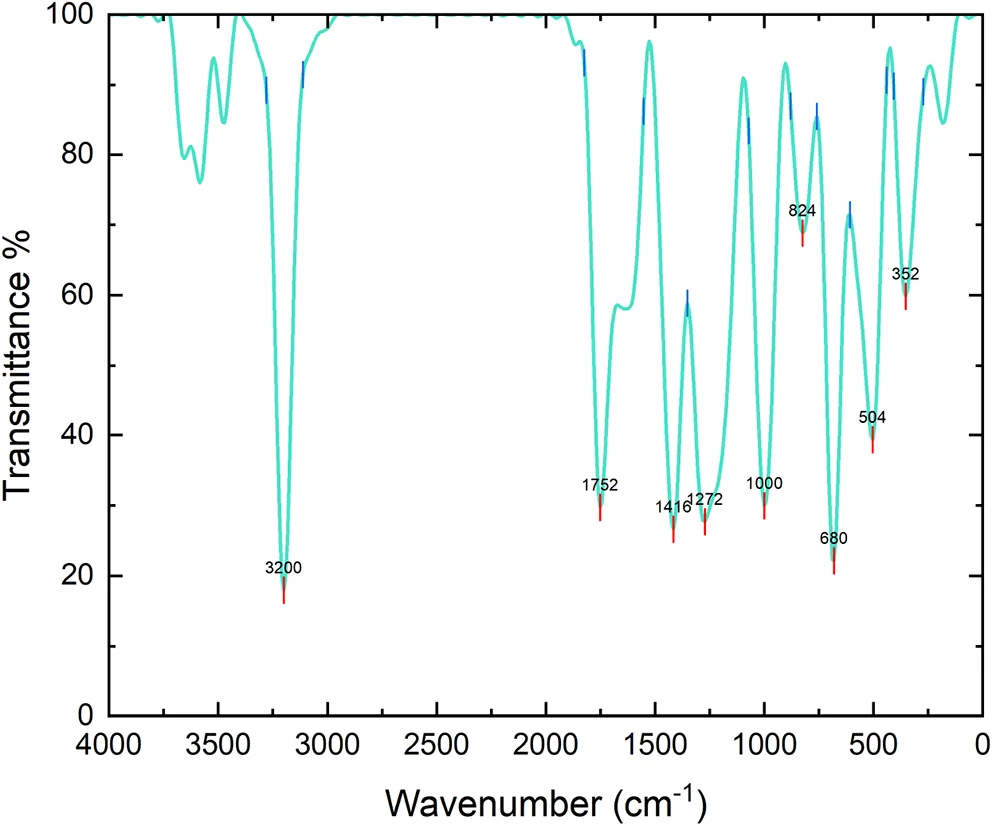
Theoretically simulated FT-IR spectrum of 2-aminosuccinic acid calculated at the B3LYP/6-311G­(d,p) level.

**Table 3:** Selected Calculated Vibrational Frequencies and Their Assignments for 2-Aminosuccinic Acid

frequency (cm^‑1^)	intensity (km/mol)	assignment	functional group
∼3000	very strong	ν(O–H), ν(N–H)	carboxyl O–H and amino N–H
1742	strongest	ν(C=O)	carbonyl (C=O) stretch
1450–1400	medium	δ(O–H), δ(C–O–H)	carboxyl in-plane bending
1350–1250	medium/strong	ν(C–O)	C–OH stretch
1200–1100	medium	ν(C–C), ν(C–N)	backbone stretching
<1000	variable	δ(C–H), skeletal deformations	aliphatic bends and ring/chain deformations

#### 3.3.2 Nuclear Magnetic Resonance Spectroscopy

Nuclear magnetic resonance (NMR) spectroscopy is a powerful tool for explaining the molecular structure by probing the local electronic environment of specific nuclei. To complement the structural findings from geometry optimization, theoretical ^1^H and ^13^C NMR chemical shifts of 2-aminosuccinic acid were calculated at the B3LYP/6-311G­(d,p) level using the gauge-independent atomic orbital (GIAO) method. The predicted spectra are presented in Figure 4, and the computed chemical shifts are detailed in Table 4 with assignments made based on the electronic environment of each nucleus.

**Figure 4:**
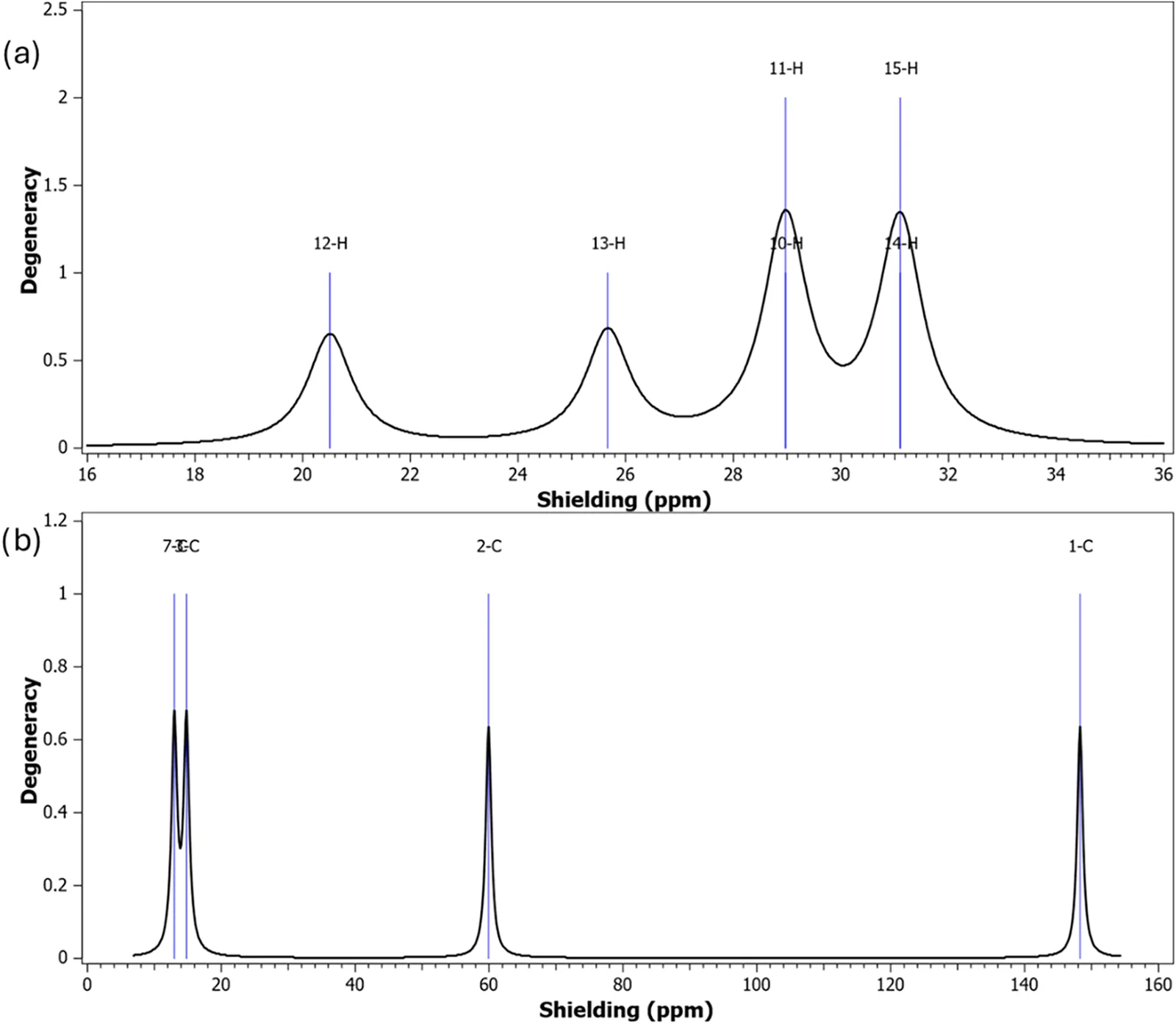
Theoretically simulated (a) ^1^H NMR and (b) ^13^C NMR spectra of 2-aminosuccinic acid.

**Table 4:** Calculated ^1^H and ^13^C NMR Chemical Shifts (δ, ppm) for 2-Aminosuccinic Acid

nucleus type	atom label	calculated δ (ppm)	assignment/chemical environment
1H	H12, H13 (COOH)	11.8	carboxylic acid protons
1H	H14, H15, H16 NH_3_^+^	6.5–7.5	protons of the protonated amino group
1H	H10, H11, etc. (CH, CH_2_)	2.0–3.0	aliphatic backbone protons
13C	C7, C3 (C=O)	175.5, 172.0	carbonyl carbons of carboxyl groups
13C	C2 (C–NH_3_^+^)	48.0	methine carbon bonded to the amino group
13C	C1, etc. (CH_2_, CH)	35–40	other aliphatic backbone carbons

#### 3.3.3 UV–Visible Analysis

Ultraviolet–visible (UV–vis) spectroscopy provides critical insights into the electronic structure of molecules by probing electronic transitions from the ground state to excited states. To investigate the optical properties and electronic excitations of 2-aminosuccinic acid, TD-DFT calculations were performed at the B3LYP/6-311G­(d,p) level in the gas phase. The simulated absorption spectrum, along with the corresponding oscillator strengths, is presented in Figure 5. The simulated spectrum (Figure 5) reveals that 2-aminosuccinic acid shows strong absorption exclusively in the deep ultraviolet (UV) region, with no significant absorption bands seen in the visible range (400–800 nm). This result follows the colorless nature of the compound. The primary absorption occurs as an intense band centered at about λ ≈ 165 nm, with more, weaker features at slightly higher wavelengths.

**Figure 5:**
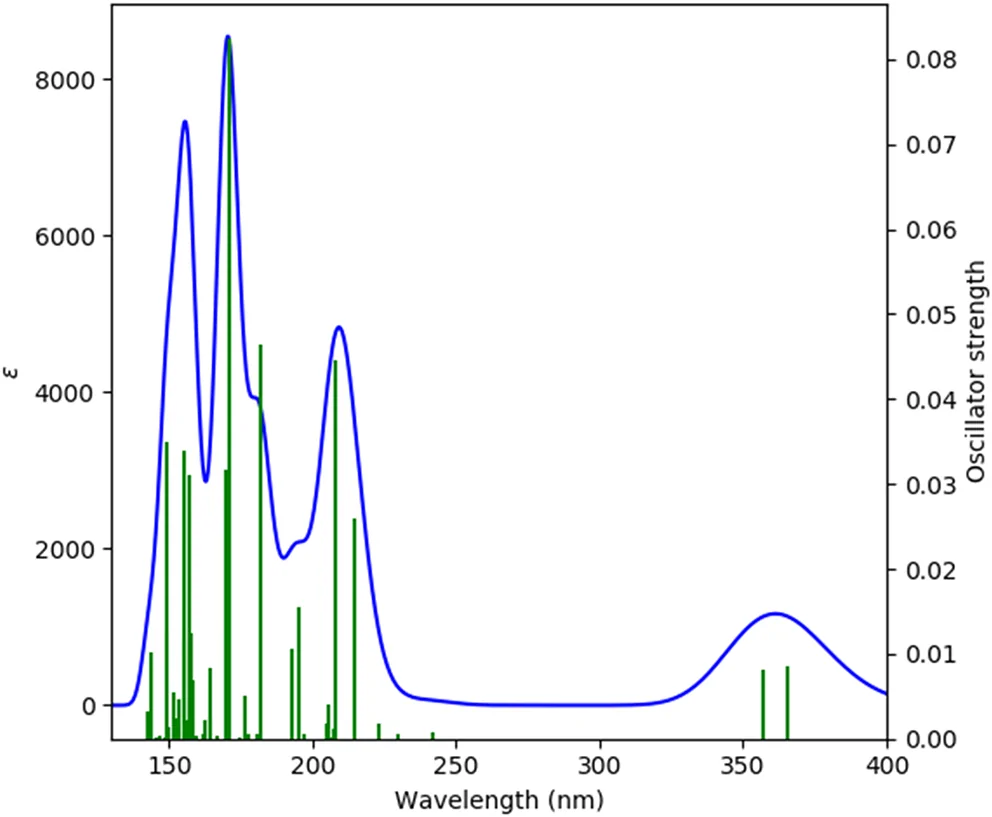
Theoretically simulated UV–vis absorption spectrum of 2-aminosuccinic acid. Vertical bars represent the calculated excitation energies and their corresponding oscillator strengths.

#### 3.3.4 Comparison with Experimental Literature Data

To strengthen the reliability of the simulated spectroscopic results, the calculated FT-IR, NMR, and UV–vis features of 2-aminosuccinic acid were compared with available experimental literature data for aspartic acid. No new experimental spectroscopic measurements were performed in this study. Instead, experimentally reported spectral features were used as reference data because aspartic acid is a well-known amino acid with documented FT-IR, Raman, and NMR spectral information in the literature. This comparison was included to confirm the present theoretical predictions and clarify the degree of agreement between the calculated and experimentally observed spectral behaviors.

The calculated FT-IR spectrum reproduces the main vibrational features of aspartic acid, especially the carbonyl-/carboxylate-related vibrations and the broad hydrogen-bond-influenced stretching region. Experimental FT-IR studies have reported that the vibrational behavior of solid aspartic acid is affected by its zwitterionic structure and hydrogen-bonding network. The absence or strong broadening of conventional O–H and N–H stretching bands in the expected spectral region has been associated with zwitterionic stabilization and intermolecular hydrogen bonding. The calculated vibrational pattern in the present study therefore follows the seen experimentally hydrogen-bond-dominated spectral behavior of aspartic acid.

The calculated NMR chemical shifts also show reasonable agreement with the experimental NMR data reported for l-aspartic acid in an aqueous solution. Experimental metabolomics databases report ^1^H NMR spectra of l-aspartic acid recorded in water or D_2_O under physiologically like conditions, while BMRB provides assigned chemical shift data at pH 7.4. The calculated aliphatic proton and carbonyl carbon regions follow the expected spectral ranges of aspartic acid, although differences are expected because the present calculations were performed using an isolated-molecule theoretical model, but experimental NMR spectra are affected by the solvent, pH, protonation state, and intermolecular interactions.

For UV–vis properties, the simulated spectrum indicates dominant absorption in the deep ultraviolet region and no significant absorption in the visible range. This result follows the colorless nature of aspartic acid and the absence of an extended conjugated chromophore. Therefore, the theoretical UV–vis result supports the expected optical behavior of the molecule. Comparison with experimental literature data confirms that the simulated spectroscopic results are chemically reasonable and suitable for supporting the structural characterization of 2-aminosuccinic acid.

### 3.4 Molecular Electrostatic Potential (MEP) Analysis

Molecular electrostatic potential (MEP) analysis is used to visualize the charge distribution and identify the most reactive sites for electrophilic and nucleophilic attacks within the molecule. As depicted in Figure 6, the MEP map of 2-aminosuccinic acid displays distinct color-coded regions based on electrostatic potential values. The regions with negative electrostatic potential, typically in red or orange, are concentrated around the oxygen atoms of the carboxyl groups, suggesting electron-rich sites that are susceptible to electrophilic interactions. The regions with positive electrostatic potential, typically in blue, are located around the hydrogen atoms attached to the oxygen and nitrogen, representing electron-deficient sites that are prone to nucleophilic attack. This mapping provides important insights into the intermolecular interactions and hydrogen-bonding capability of the molecule.

**Figure 6:**
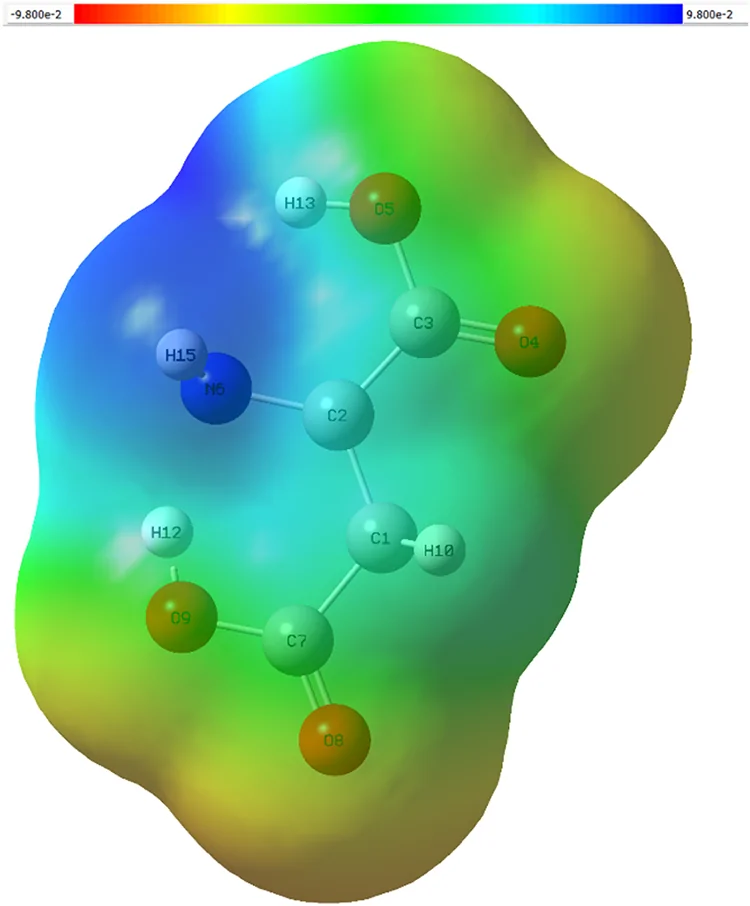
Molecular electrostatic potential (MEP) map of 2-aminosuccinic acid. The surface is color-coded on an electrostatic potential scale from red (most negative, −0.06 au) to blue (most positive, +0.06 au).

### 3.5 Density of States (DOS) and Total Density of States (TDOS)

The density of states (DOS) and total density of states (TDOS) analyses provide a detailed and quantitative perspective on the electronic structure of a molecule by projecting the contributions of molecular orbitals (MOs) onto specific atoms or fragments. This analysis complements the frontier orbital (FMO) picture by revealing the composition and energy distribution of all occupied and virtual orbitals involved in electronic transitions. The DOS and TDOS spectra for 2-aminosuccinic acid were generated using the Multiwfn program based on the B3LYP/6-311G­(d,p) calculated wave function and are presented in Figure 7. The TDOS plot (Figure 8) illustrates the overall distribution of electronic energy levels. A clear energy gap between the highest occupied and lowest unoccupied states is visible, corresponding to the HOMO–LUMO gap of 3.308 eV calculated earlier. The valence band (occupied orbitals) shows several dense regions corresponding to groupings of orbitals with similar energies, primarily originating from the σ- and π-bonding networks of the carboxyl and amino groups.

**Figure 7:**
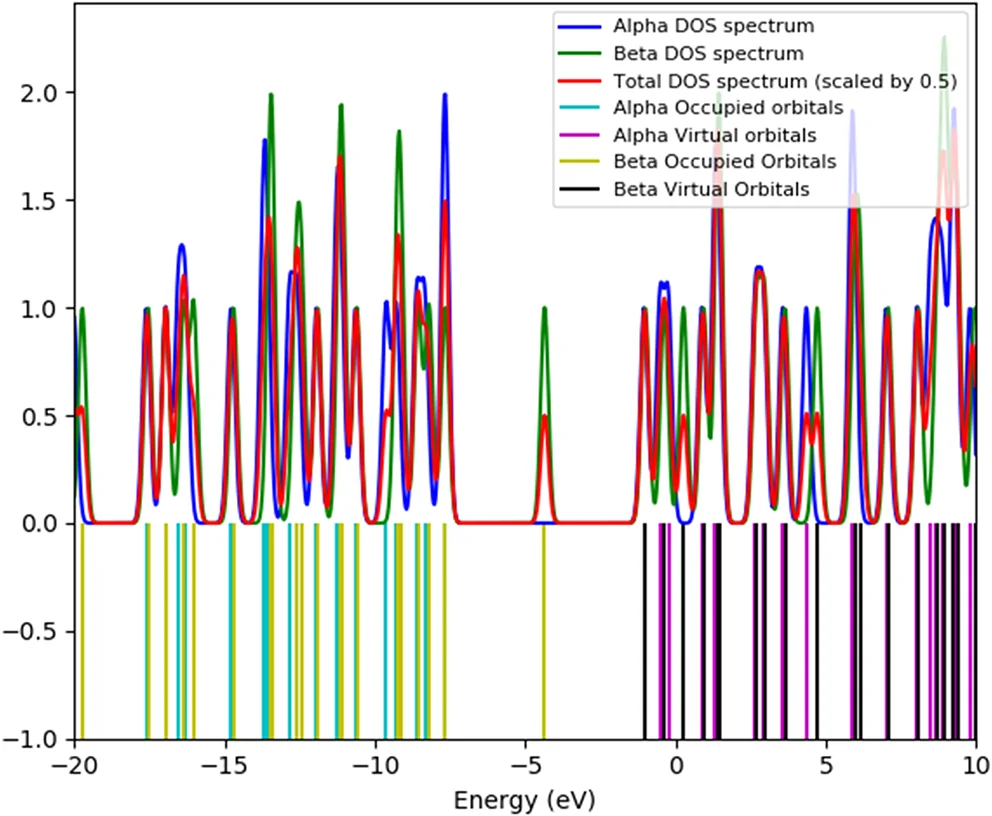
Density of states (DOS) of 2-aminosuccinic acid.

**Figure 8:**
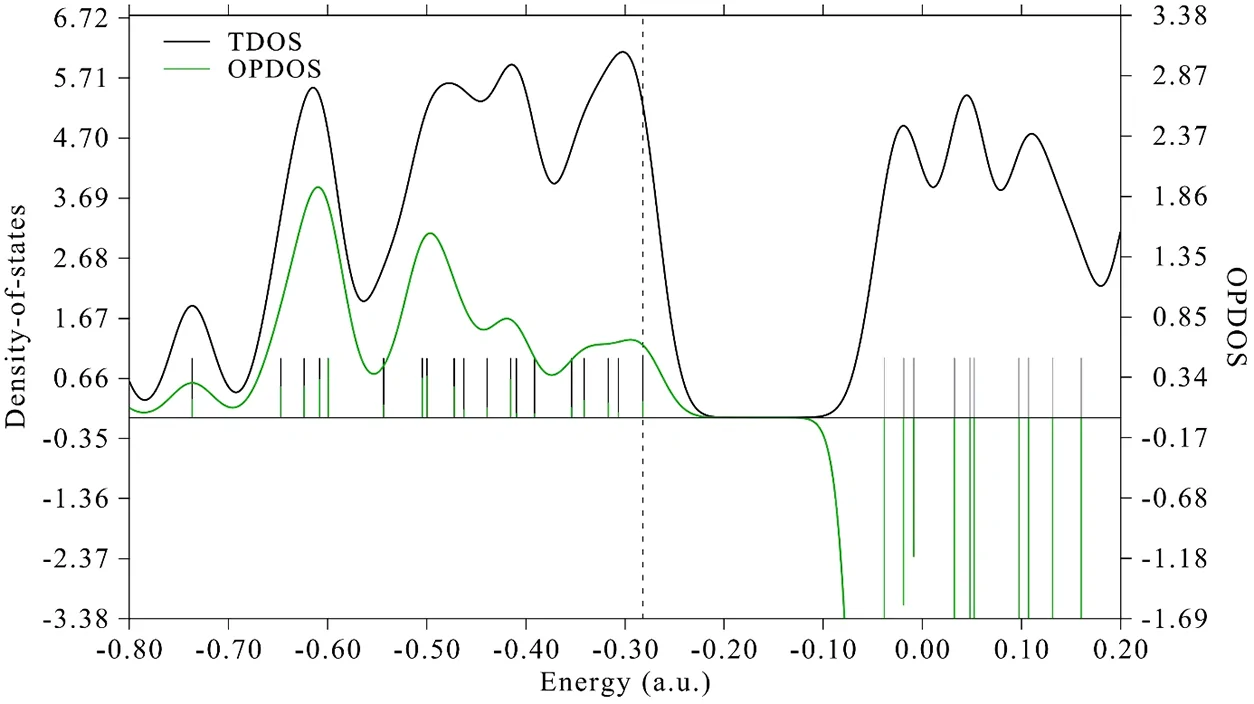
Total density of states (TDOS) of 2-aminosuccinic acid.

### 3.6 Non-Covalent Interaction (NCI) and DORI Analysis

Non-covalent interactions (NCIs) such as hydrogen bonds, van der Waals forces, and steric repulsions are pivotal in determining the molecular conformation, stability, and intermolecular assembly. To visualize and characterize these subtle yet critical forces within 2-aminosuccinic acid, combined non-covalent interaction (NCI) and domain-based local pair natural orbital (DORI) analyses were performed. The results, visualized through NCI isosurfaces and DORI plots in Figure 9, provide a three-dimensional map of the attractive and repulsive interactions governing the molecule’s structure. The NCI analysis (Figure 9a) uses the reduced density gradient (RDG) plotted against the sign­(λ_2_)­ρ function, where ρ is the electron density and λ_2_ is the second eigenvalue of its Hessian. This is visualized both as an isosurface and a 2D scatter plot (Figure 9c).

**Figure 9:**
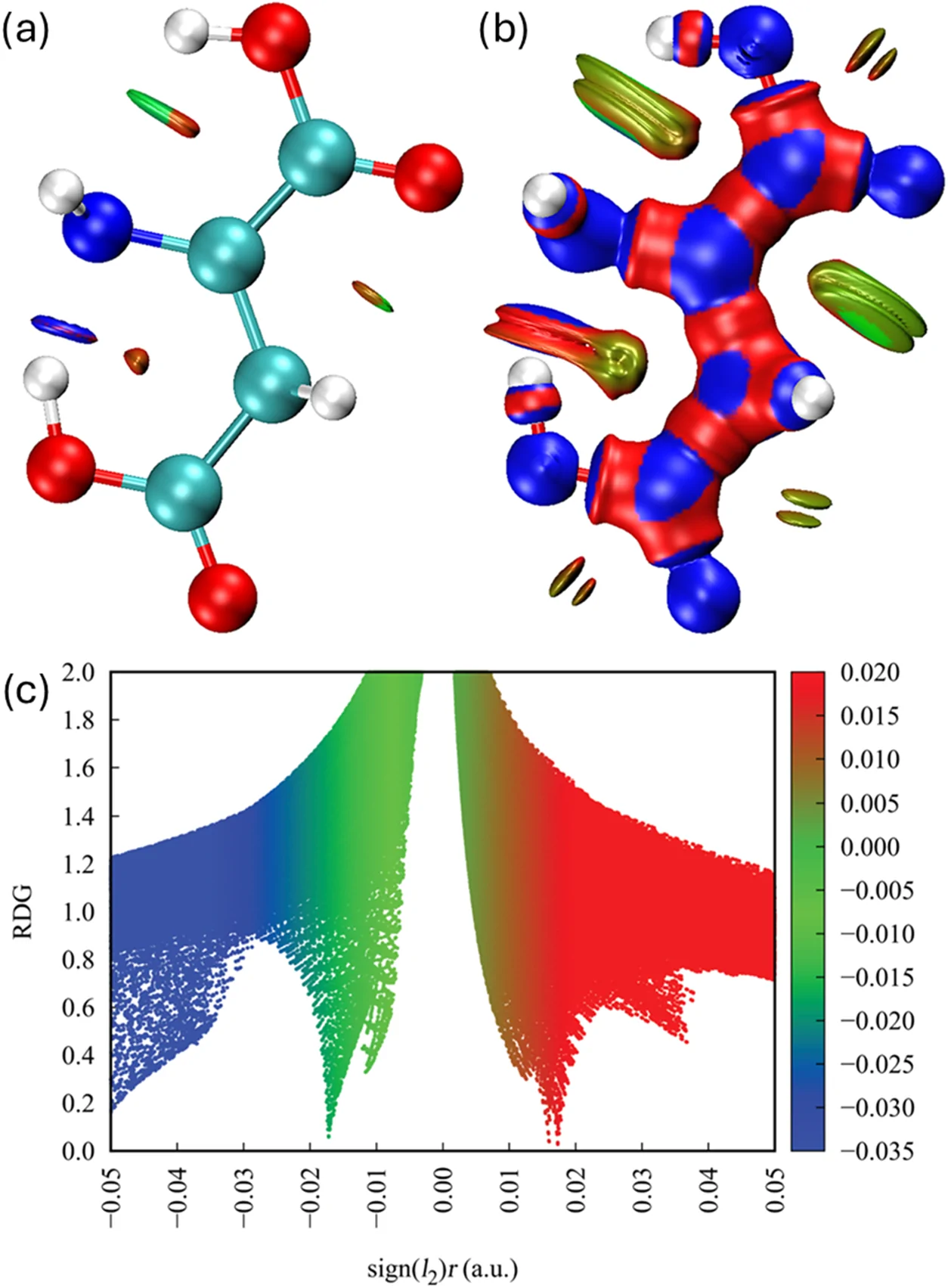
(a) NCI, (b) DORI, and (c) RDG for 2-aminosuccinic acid.

### 3.7 Thermo-Chemistry Map (TCM)

To evaluate the thermodynamic stability and temperature-dependent behavior of 2-aminosuccinic acid, thermodynamic parameters including thermal energy (*E*), heat capacity at constant volume (*C_v_*), and entropy (*S*) were calculated over the 300–900 K temperature range. Figure 10 presents a molecular surface representation associated with the thermochemical characterization of 2-aminosuccinic acid, but Figure 11 and Table 5 provide the quantitative temperature-dependent thermodynamic results. As shown in Figure 11, the molar thermal energy goes up progressively with temperature with total, translational, rotational, and vibrational contributions presented separately. The results indicate that as the temperature goes up from 300 to 900 K, the calculated thermodynamic parameters exhibit a consistent upward trend. Specifically, the total thermal energy and entropy rise steadily because of enhanced molecular motion and an increased population of higher-energy vibrational states. The heat capacity also increases with temperature, mainly because of the growing contribution of vibrational modes at elevated temperatures. The detailed thermodynamic data calculated at selected temperatures are summarized in Table 5a–5d. Table 5a presents the total thermodynamic parameters, while Table 5b, 5c, and 5d separately provide the translational, rotational, and vibrational contributions, respectively. These values provide useful insights into the thermochemical behavior of 2-aminosuccinic acid under different thermal conditions.

**Figure 10:**
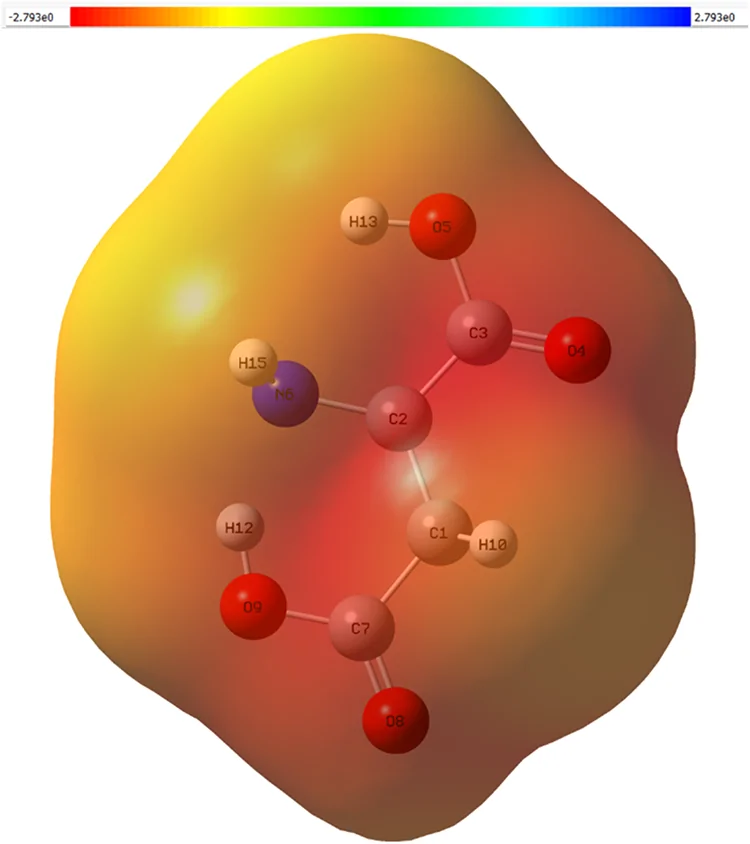
Thermochemical surface representation of 2-aminosuccinic acid generated to visualize the spatial distribution of thermochemistry-related molecular features.

**Figure 11:**
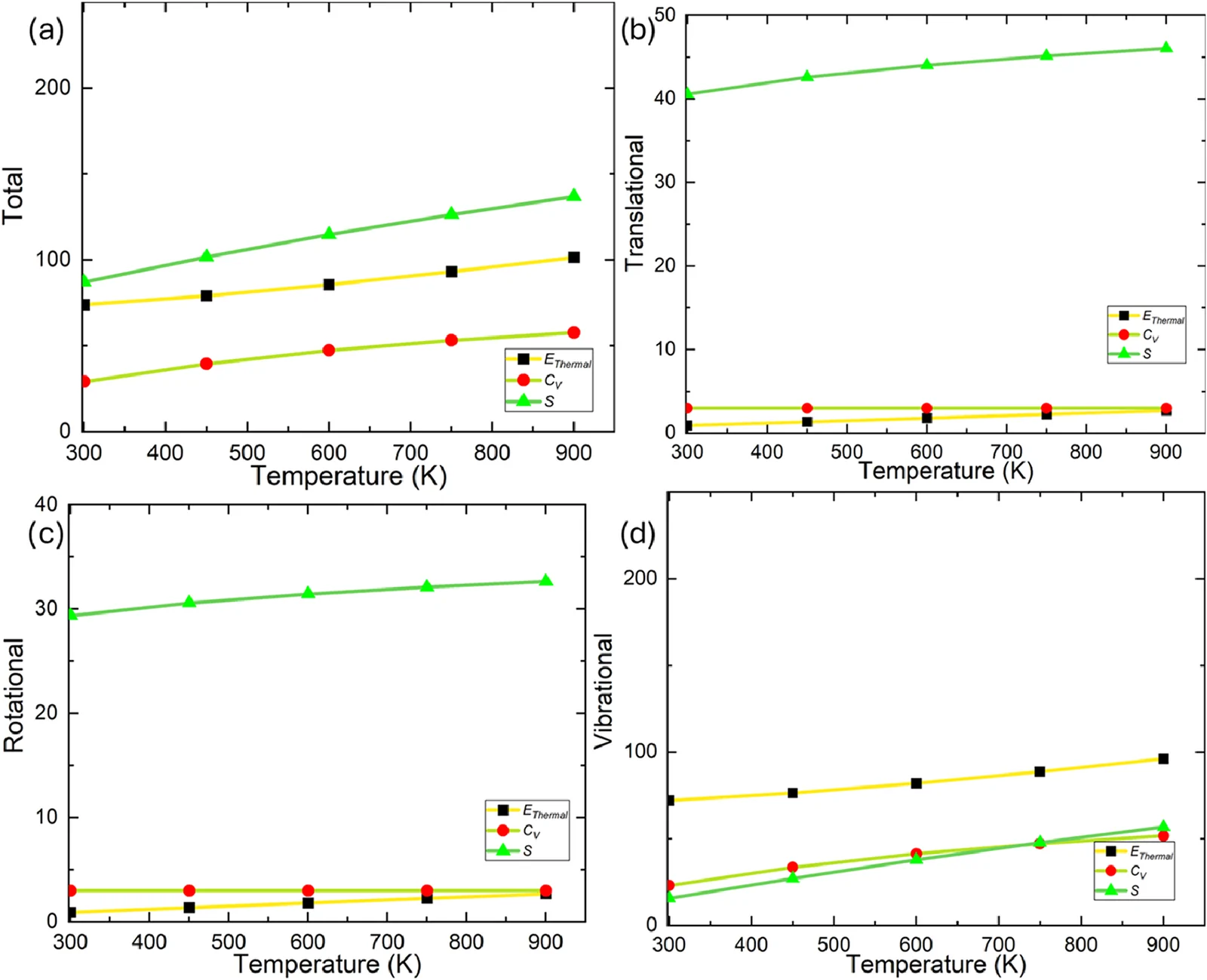
Contributions to the molar thermal energy of 2-aminosuccinic acid: (a) total, (b) translational, (c) rotational, and (d) vibrational parts as a function of temperature.

**Table 5:** (a) Total Thermodynamic Parameters of 2-Aminosuccinic Acid at Selected Temperatures; (b) Translational Contributions to the Thermodynamic Parameters of 2-Aminosuccinic Acid at Selected Temperatures; (C) Rotational Contributions to the Thermodynamic Parameters of 2-Aminosuccinic Acid at Selected Temperatures; and (D) Vibrational Contributions to the Thermodynamic Parameters of 2-Aminosuccinic Acid at Selected Temperatures

temperature (K)	*E* (kcal mol^–1^)	*C_v_* (cal mol^–1^ K^–1^)	*S* (cal mol^–1^ K^–1^)
(a) Total			
300	73.879	29.045	87.029
450	79.056	39.495	101.685
600	85.595	47.311	114.744
750	93.152	53.174	126.405
900	101.481	57.697	136.878
(b) Translational			

300	0.894	2.981	40.577
450	1.341	2.981	42.591
600	1.788	2.981	44.021
750	2.236	2.981	45.129
900	2.683	2.981	46.035
(c) Rotational			

300	0.894	2.981	29.363
450	1.341	2.981	30.572
600	1.788	2.981	31.430
750	2.236	2.981	32.095
900	2.683	2.981	32.638
(d) Vibrational Contributions to the Thermodynamic Parameters of 2-Aminosuccinic Acid at Selected Temperatures			

300	72.091	23.084	15.712
450	76.373	33.533	27.144
600	82.018	41.350	37.917
750	88.680	47.213	47.803
900	96.115	51.735	56.828

### 3.8 Hirshfeld Surface

Hirshfeld surface analysis was performed using the experimental CIF file of dl-aspartic acid obtained from the Crystallography Open Database (COD entry 2215349). Therefore, the calculated fingerprint plots and contact percentages represent intermolecular interactions in the experimentally determined solid-state crystal packing environment. To quantitatively assess the intermolecular interactions that govern the crystal packing and stability of 2-aminosuccinic acid, a Hirshfeld surface analysis was performed (Figure 12). This method partitions the crystal space into regions where the electron distribution of a molecule dominates over the sum of the electron distributions of all other molecules. The resulting Hirshfeld surfaces and their associated two-dimensional fingerprint plots provide an unambiguous, visual summary of all close intermolecular contacts. The fingerprint plot (Figure 12a) depicts the distribution of distances from points on the Hirshfeld surface to the nearest external (de) and internal (di) nuclei. The shape and density of the plot reveal the nature of dominant contacts. For 2-aminosuccinic acid, the plot is characterized by two sharp spikes extending toward the bottom left region (di > de), which are hallmark signatures of strong hydrogen-bonding interactions.

**Figure 12:**
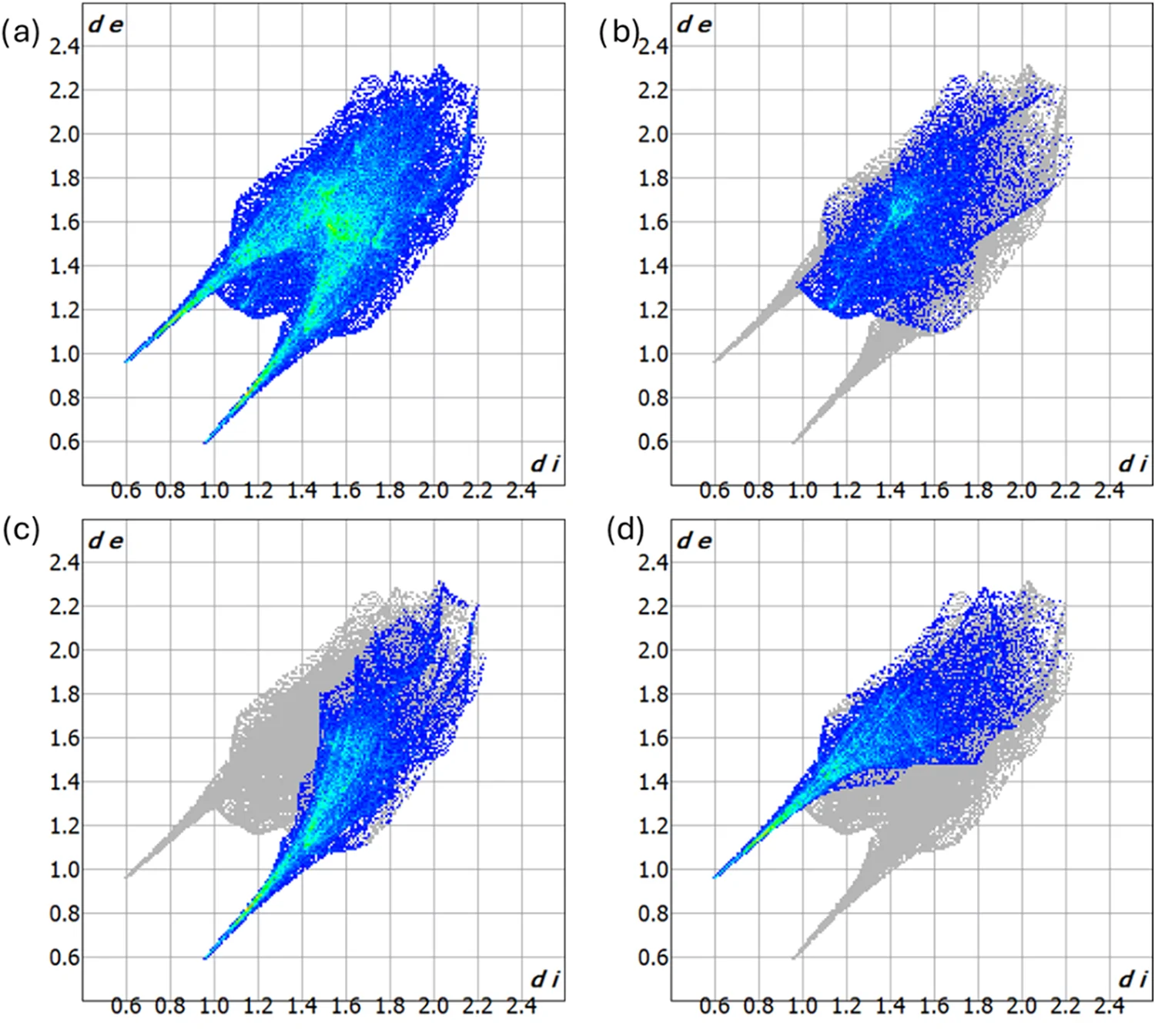
Hirshfeld surface analysis of 2-aminosuccinic acid: (a) full two-dimensional fingerprint plot; (b) H···H contacts contributing 23.0%; (c) O···H contacts contributing 35.1%; and (d) H···O contacts contributing 29.8%.

### 3.9 Molecular Docking

Instead of using the traditional ″drug-target inhibition″ method, the possible biological effects of 2-aminosuccinic acid in the setting of cancer were assessed in this study using metabolic and redox-based mechanisms. Instead of functioning as a strong and specific enzyme inhibitor, the molecule may indirectly affect cellular metabolic fluxes and redox balance because of its tiny size, highly polar nature, and metabolic intermediate properties. The SLC1A1, SLC7A11, and glutathione (GSH) axis, which is intimately linked to metabolic adaptability and ferroptosis in cancer cells was the focus of molecular docking studies. SLC1A1 (EAAT3) is a transporter that helps fulfill increasing metabolic needs, particularly in cancer cells that are growing quickly. It is involved in the intracellular transfer of acidic amino acids such aspartate and glutamate.^41^ With a binding energy of −4.6 kcal/mol and a large number of hydrogen bonds, the interaction profile of 2-aminosuccinic acid with SLC1A1 in this work indicates that the molecule can be identified by this carrier in a way similar to that of a substrate. Given the low-to-medium affinity and inherent transience of carrier protein–substrate interactions, this binding energy is biologically substantial and supports the possibility of active transport of the chemical into the cell. By generating the light chain of the cystine/glutamate antiporter system, SLC7A11 (xCT), an important ferroptosis determinant, controls the absorption of cystines into the cell and the production of glutathione.^22^ It is well-known that cancer cells often express more SLC7A11, and that this expression is linked to resistance to treatment and tolerance to oxidative stress.^24^ With a binding energy of −5.0 kcal/mol and six hydrogen bonds, 2-aminosuccinic acid’s interaction with SLC7A11 in this study points to a temporary interaction that might fine-tune the transporter function rather than a potent inhibitory effect (Table 6). Cysteine uptake and, indirectly, glutathione biosynthesis may be affected by this interaction, which could weaken the redox balance of cancer cells. A key player in the detoxification of reactive oxygen species and the inhibition of lipid peroxidation, glutathione (GSH) is an important element of cellular redox homeostasis.^44^ In this investigation, the molecule’s chemical compatibility with intracellular redox parts is shown by the binding energy of 2-aminosuccinic acid with GSH, which shows a dense hydrogen-bond network and a binding energy of −4.9 kcal/mol. These interactions imply that GSH may indirectly affect the glutathione pool or redox microenvironment, despite not being a ″target protein″ in the traditional sense. Evaluating the docking results with SLC1A1, SLC7A11, and GSH combined yields a coherent model of metabolic-redox interaction (Table 6). In this scenario, 2-aminosuccinic acid is mostly absorbed via amino acid transporters, which cause precise changes in glutathione-dependent antioxidant defense mechanisms, making cancer cells more vulnerable to ferroptosis and oxidative stress. Instead of immediately producing cytotoxicity, this mechanism of action is believed to have a ″sensitizing″ effect that restricts the ability of cancer cells to adapt metabolically and redoxically. These results might eventually enable the assessment of 2-aminosuccinic acid as a metabolic adjuvant that can be combined with ferroptosis inducers or traditional chemotherapeutics, rather than as a stand-alone anticancer drug. The potential of these tiny metabolites to overcome resistance and improve treatment sensitivity in cancer therapy justifies more exploration, given the growing significance of treatment approaches that target amino acid transporters and glutathione metabolism.

**Table 6:**
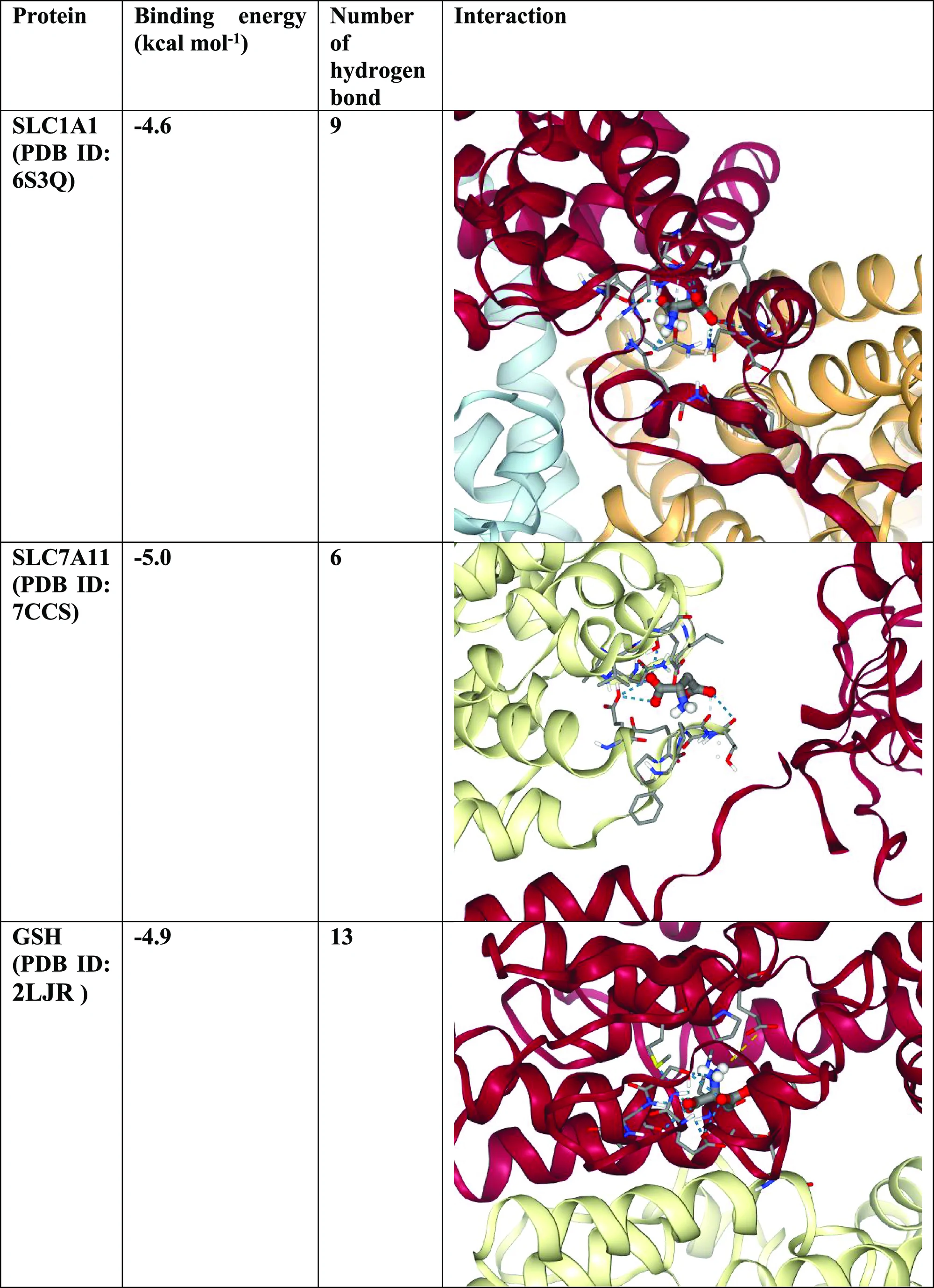
Molecular Docking Results of 2-aminosuccinic Acid with Selected Human SLCA1, SLC7A11, and GSH Proteins

## 4 Conclusion

Through extensive DFT-based quantum-chemical analyses, this study reveals the molecular structure, electronic features, spectroscopic nature, and thermodynamic behavior of 2-aminosuccinic acid (aspartic acid). A molecular docking approach is also used to assess the molecule’s potential for interaction with biologically significant targets. The findings explain the molecule’s functional features and physicochemical stability, which allow it to interact with biological systems. According to DFT calculations, 2-aminosuccinic acid has a HOMO–LUMO energy gap of 3.308 eV, which gives it kinetic stability and enough reactivity for biological interactions. It is also highly stable in its zwitterionic form and is maintained by strong intramolecular hydrogen bonds. The behavior of the molecule is mostly determined by intramolecular and intermolecular contacts, and oxygen-rich functional groups are receptive to hydrogen bonding, according to molecular electrostatic potential (MEP), NCI, and Hirshfeld surface investigations. The molecule’s capacity to adjust to protein binding sites is supported by these features. In support of this theoretical approach, molecular docking analyses have shown that 2-aminosuccinic acid has thermodynamically substantial binding affinities with the GPX4/GSH system, SLC1A1 (EAAT3), and SLC7A11 (xCT), three important molecular targets linked to ferroptosis. As for controlling the intracellular cysteine/glutathione balance and changing ferroptosis sensitivity, the strong binding seen with SLC7A11 is remarkable. These interactions are essential to the molecule’s binding to biological targets, as shown by the many hydrogen bonds seen at the docking locations, which are in line with the hydrogen-bonding tendency found in DFT and NCI investigations. The electronic structure, charge distribution, and hydrogen-bonding ability that DFT reveals offer a solid theoretical foundation for explaining the molecular-level protein–ligand interactions in docking studies. According to the results, 2-aminosuccinic acid may be viewed as a modulator that can affect ferroptosis regulation through amino acid transport and redox homeostasis rather than as a direct cytotoxic agent. This study concludes by providing a thorough and coherent theoretical profile of 2-aminosuccinic acid along the axes of structure, electrical properties, and biological interactions. This molecule and its derivatives can serve as a logical starting point for upcoming ferroptosis-targeted cancer therapeutics, medicinal chemistry applications, and biomolecular design research, as shown by the integrated DFT and molecular docking techniques that were given. These findings offer a solid point of reference for upcoming experimental validation research. The DFT results obtained in the gas phase represent intrinsic molecular descriptors; therefore, future studies including explicit or implicit aqueous solvation models and different protonation states would provide a more physiologically relevant description of 2-aminosuccinic acid.

## Data Availability

Data is provided within the manuscript. The data underlying this article will be shared on reasonable request to the corresponding author.
